# Analysis of Isoflavones in Pueraria by UHPLC-Q-Orbitrap HRMS and Study on α-Glucosidase Inhibitory Activity

**DOI:** 10.3390/foods11213523

**Published:** 2022-11-05

**Authors:** Yan Yang, Hui Zhao, Furong Zhu, Xiaoyan Liu, Yu Liu, Feng Zeng, Bin Liu

**Affiliations:** 1 College of Food Science, Fujian Agriculture and Forestry University, Fuzhou 350002, China; 2 Department of Physical and Chemical Analysis, Fujian Center for Disease Control and Prevention, Fuzhou 350001, China; 3 College of Life Sciences, Fujian Agriculture and Forestry University, Fuzhou 350002, China; 4 Beijing Engineering and Technology Research Center of Food Additives, School of Food and Health, Beijing Technology and Business University, Beijing 100048, China

**Keywords:** *Pueraria lobata*, UHPLC-Q-Orbitrap HRMS, isoflavone, molecular docking, α-glucosidase

## Abstract

Pueraria is a rich source of bioactive compounds, but there is a lack of comprehensive information concerning its composition. Therefore, a UHPLC-Q-Orbitrap HRMS method was developed to identify and quantify bioactive compounds in pueraria. Twelve isoflavones were quantified, with puerarin being the most abundant, followed by puerarin 6″-O-xyloside, 3′-methoxy puerarin, and 3′-hydroxy puerarin. A further 88 bioactive components in eight categories were also tentatively identified. The 12 isoflavones, except for genistein, exhibited α-glucosidase inhibitory activity. The binding of these compounds to the active site of α-glucosidase was confirmed via molecular docking analysis. These findings provide a basis for identifying pueraria as a promising functional food ingredient.

## 1. Introduction

Pueraria is the dried root of *Pueraria lobata* (Willd.) Ohwi and is used as a functional food in countries in East Asia [1]. It contains several bioactive components (such as puerarin, daidzein, genistein, and various steroids) that possess an extensive range of pharmacological properties including cardioprotective, neuroprotective, antioxidative, anti-inflammatory, antidiabetic, hepatoprotective, hypolipidemic, and antiosteoporosis effects [2,3]. Many studies show that pueraria extracts have antidiabetic activity [4,5,6,7,8,9], however, the active ingredients in these extracts are unknown.

Pueraria research has focused on determining its puerarin content and pharmacological properties [10,11]. Some studies have demonstrated that the antidiabetic properties of pueraria are dependent not only on puerarin but also on minor isoflavonoids [1,12] including 3′-hydroxy puerarin, 3′-methoxy puerarin, puerarin 6″-O-xyloside, formononetin, and ononin [13].

The inhibition of α-glucosidase activity can decrease the rate of blood sugar absorption and improve insulin sensitivity, thus affecting glucose levels [14,15].α-Glucosidase inhibition also has significant effects on polysaccharide metabolism and glycoprotein processing [16]. Many flavones, such as apigenin-7-O-glucoside, calycosin, and isoquercetin [17], have recently been identified as potential α-glucosidase inhibitors, and considerable effort has been applied to isolate useful inhibitors from natural food sources to develop functional foods against diabetes [18].

Further research is needed into the composition and α-glucosidase inhibition mechanisms of the active ingredients of pueraria, and a method for rapidly identifying and quantifying these ingredients is required. Due to complex active substance in pueraria, it is still a great challenge to establish a sensitive and accurate method of simultaneous quantification of compound in pueraria. Several methods have previously been developed for the analysis of pueraria components. These include enzyme-linked immunosorbent assay (ELISA) [19,20], high-performance liquid chromatography (HPLC) [21,22], and liquid chromatography-mass spectrometry (LC-MS/MS) [23,24]. However, most of these methods are limited to the determination of only 3 to 6 compounds. In recent years, UHPLC coupled with hybrid quadrupole-Orbitrap high resolution tandem mass spectrometry (UHPLC-Q-Orbitrap HRMS) has emerged as a new technology capable of analyzing the active ingredients in food products [25,26]. The UHPLC-Q-Orbitrap HRMS system can simultaneously analyze a potentially unlimited number of compounds because its full MS-ddMS_2_ scan mode allows for the screening and quantifying of analytes and the retrospective analysis of unknown compounds [27]. Furthermore, this system provides accurate, exact mass measurements and improved selectivity and sensitivity for the detection and identification of low concentration analytes [28].

**Figure 1:**
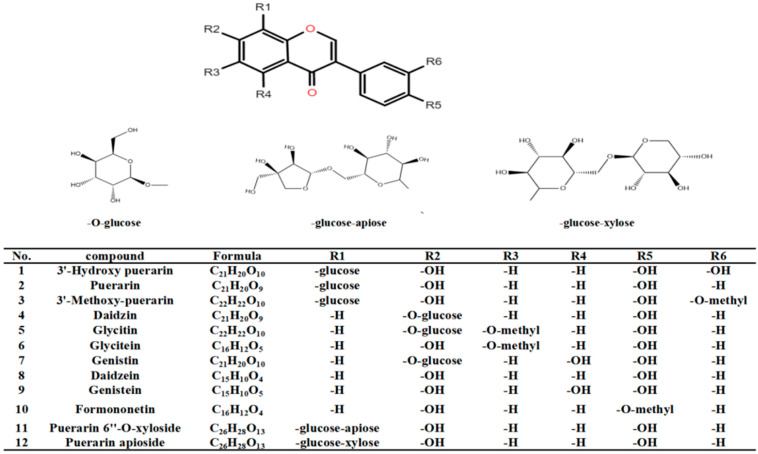
Chemical structures of the 12 isoflavone compounds.

This study aims to elucidate the bioactive compounds and mechanisms of α-glucosidase inhibition found in pueraria. A method is developed to identify and quantify the active ingredients in pueraria using UHPLC-Q-Orbitrap HRMS. The contents of 12 isoflavones in pueraria were determined. The chemical structures of the 12 isoflavone compounds are shown in Figure 1. The α-glucosidase inhibitory activity of 12 active substances is evaluated and validated by molecular docking to provide an in-depth understanding of the antidiabetic properties of pueraria.

## 2. Materials and Methods

### 2.1. Chemicals and Reagents

The standards daidzein (PubChem CID: 5281708), daidzin (PubChem CID:107971), puerarin (PubChem CID: 5281807), glycitin (PubChem CID: 187808), 3′-methoxy puerarin (PubChem CID: 5319485), genistin (PubChem CID: 5281377), genistein (PubChem CID: 5280961), formononetin (PubChem CID: 5280378), 3′-hydroxy puerarin (PubChem CID: 5748205), glycitein (PubChem CID: 5317750), puerarin 6″-O-xyloside (PubChem CID: 100990912), and puerarin apioside (PubChem CID: 21676217), with purity ≥ 98%, were purchased from ANPEL Laboratory Technologies Inc. (Shanghai, China). HPLC-grade methanol and acetonitrile were purchased from Thermo Fisher Scientific (Fair Lawn, NJ, USA) and formic acid (HPLC grade) from ANPEL. α-Glucosidase was purchased from Sigma-Aldrich, and acarbose and p-nitrophenyl-α-D-glucopyranoside (pNPG) from Yuanye Bio-Technology Co., Ltd. (Shanghai, China). Dimethyl sulfoxide (DMSO) was purchased from Fisher Scientific Inc. (Pittsburgh, PA). Ultra-pure water with resistivity < 18.2 MΩ cm was prepared in a Millipore Direct-Q water system (Millipore, Bedford, MA, USA). Nylon syringe filters (13 mm diameter, 0.22 μm) were purchased from ANPEL.

Twelve 1.0 mg/mL stock standard solutions were prepared in DMSO and stored at −20 °C in the dark for up to six months. A 10 µg/mL mixed working standard solution was prepared by mixing 100 µL of each stock standard solution in a final volume of 10 mL methanol/water (50:50, *v/v*). The working standard was stored at 4 °C in a brown glass bottle for up to one month.

### 2.2. Sample Preparation

Pueraria samples were purchased from Baicao Pharmaceutical Co. Ltd. (Anhui, China) and were identified by Professor Hong Qiu of Fujian Health College. Pueraria samples were oven-dried at 40 °C for 12 h, ground to a fine powder, and passed through a 180 µm sieve. Approximately 0.1 g of powder was accurately weighed and dissolved in 50 mL 90% ethanol. Extraction was performed in an ultrasonic bath (300 W, 40 kHz) at 50 °C for 60 min followed by centrifugation at 10,000× *g* for 5 min. The supernatant was diluted ten-fold with water and filtered through a 0.22 µm nylon membrane filter before analysis on the UHPLC-Q-Exactive Orbitrap HRMS.

### 2.3. UHPLC-Q-Exactive Orbitrap HRMS Analysis

A Q-Exactive Orbitrap high resolution tandem mass spectrometer coupled to a Dionex Ultimate 3000 UHPLC system (Thermo Fisher, MA, USA) equipped with a Waters Acquity UPLC BEH C_18_ column (2.1 mm × 100 mm, 1.7 μm) maintained at 40 °C was used for analysis of pueraria extracts. The flow rate was 0.3 mL/min, injection volume was 2 μL, and the mobile phase consisted of (A) acetonitrile and (B) water containing 0.2% formic acid. Electrospray ionization parameters were as follows: auxiliary gas flow 10 arb, sheath gas flow 35 arb, spray voltage 3.5 kV (positive mode) and 3.2 kV (negative mode), capillary temperature 320 °C, probe heater temperature 350 °C, and S-lens 60. Mass scan parameters were as follows: scan mode Full ms-ddms2, Full MS scan range 100–1500 m/z, resolution Full MS 70000 FWHM and MS/MS 17500 FWHM, AGC target Full MS 1e6 and MS/MS 2e5, maximum IT Full MS 100 ms and MS/MS 50 ms, Loop count 3, MSX count 1, Isolation width 1.5 m/z, NCE (Stepped) 10/30/40, minimum AGC target 8e3, Intensity Threshold 1.6e5, Dynamic exclusion 5 s.

#### 2.3.1. Quantitative Method

The UPLC gradient was as follows: 0–7.5 min 95-60% B, 7.5–11 min 60–5% B, 11-12 min 5% B, 12.1 min 5–95% B, and 12.1–15 min 95% B. Full MS-ddMS_2_ scanning was performed in positive ionization mode.

#### 2.3.2. Screening Method

The UPLC gradient was as follows: 0–2 min 95% B, 2–42 min 95–5% B, 42–47 min 5% B, 47.1 min 5–95% B, and 47.1–50 min 95% B. Full MS-ddMS_2_ scanning was performed in both positive and negative ionization modes. The Thermo Trace Finder data analysis “Screening Method″ was used to search for analytes by comparison to a compound database using exact mass measurements of precursor ions (<5 ppm), MS/MS fragmentation, LC retention time, and the chemical compounds database query.

### 2.4. Method Validation

The optimized method was validated according to AOAC International [29] for the following parameters: linearity, limit of detection (LOD), limit of quantitation (LOQ), accuracy (recovery), and precision (relative standard deviation, RSD). Linearity was evaluated for 12 compounds using eight-point calibration curves (0.1, 5, 10, 20, 50, 100, 200, and 300 μg/L), plotting peak area (y) against concentration (x), with 1/x weighting. Recovery was determined by quantifying samples fortified with known concentrations of standards, corresponding to approximately 50%, 100%, and 150% of the concentrations expected in pueraria samples (n = 6). Intra-day (repeatability) and inter-day precision (reproducibility) were determined by the repeated analysis of samples (n = 6) fortified at three concentrations and tested on the same day (intra-day) or on three consecutive days (inter-day). Precision was expressed as the relative standard deviation (RSD) for each compound at each concentration. LOD and LOQ were estimated by diluting standard solutions to the lowest detectable concentrations and calculating three and ten times the signal-to-noise ratio, respectively.

### 2.5. α-Glucosidase Inhibitory Activity

α-Glucosidase inhibitory activity was assessed as previously described [15,30], with minor modifications. Twelve different isoflavone standards (500, 250, 125, 62.5, and 31.25 mg/L), α-glucosidase (0.2 U/mL), and 4-nitrophenol-α-D-glucopyranoside (*p*NPG, 50 μM) were each dissolved in phosphate buffer (PBS, pH 6.8). Acarbose was used as a positive control. Test solution (30 μL) and α-glucosidase (30 μL) were placed to a 96-well plate and incubated at 37 °C for 15 min. *p*NPG (30 μL) was then added to the wells and incubated at 37 °C for a further 20 min before quenching the reaction with 100 μL of 0.1 mol/L Na_2_CO_3_ and measuring the absorbance at 405 nm. Samples were analyzed in triplicate. The inhibition rate (%) was defined as $\frac{\left(A_{0}-A_{S}\right)}{A_{0}}\times 100$, where A_0_ is the absorbance of a negative control where the test solution was replaced by 30 μL PBS, and A_s_ is the absorbance of test samples. IC_50_ was defined as the half-maximal inhibitory concentration of the inhibiting compound.

### 2.6. Molecular Docking

AutoDock 4.2 Tools were used to simulate the interaction sites between the isoflavones and α-glucosidase. The 3D protein structure of α-glucosidase (PDB code: 2qmj) was used as the molecular target and downloaded from the protein database (https://rcsb.org/, accessed on 5 July 2022). Water and ligand were removed from the receptor to obtain a stable structure. The 2D structures of the 12 isoflavones were downloaded from PubChem and configured to minimize energy using Chem3D 19.0 software. PyMol software was applied to visualize the interaction processes between receptor and ligands.

## 3. Results and Discussion

### 3.1. Optimization of Chromatographic Separation and Mass Spectrometric Detection

This study analyzed full MS-ddMS_2_ scan in both positive and negative ionization modes to produce a sensitive and reliable quantitative technique. The responses of the 12 isoflavones were higher in positive mode than in negative mode. All the compounds generated a molecular ion [M + H]^+^. The product ion [M + H − 120]^+^ was observed with the C-glycosidic isoflavones (3′-hydroxy puerarin, puerarin, and 3′-methoxy puerarin) and was attributed to the loss of C_4_H_8_O_4_. The O-glycosidic isoflavones (glycitin, daidzin, and genistin) generated the prominent characteristic ion [M + H − 162]^+^ (attributed to loss of a glucose chain). The oxygen-carbon link between the glucose chain and the aglycones in O-glycosidic isoflavones was easier to break than the carbon-carbon link between glucose and aglycones in C-glycosidic isoflavones. Puerarin 6″-O-xyloside and puerarin apioside produced similar characteristic ions due to their structural similarity. Several of the isoflavones yielded the product ions [M + H − 28]^+^, [M + H − 44]^+^, [M + H − 18]^+^, or [M + H − 15]^+^ due to the loss of CO, CO_2_, H_2_O, or CH_3_. Detailed MS parameters for the 12 isoflavones are listed in Table 1.

**Figure 2:**
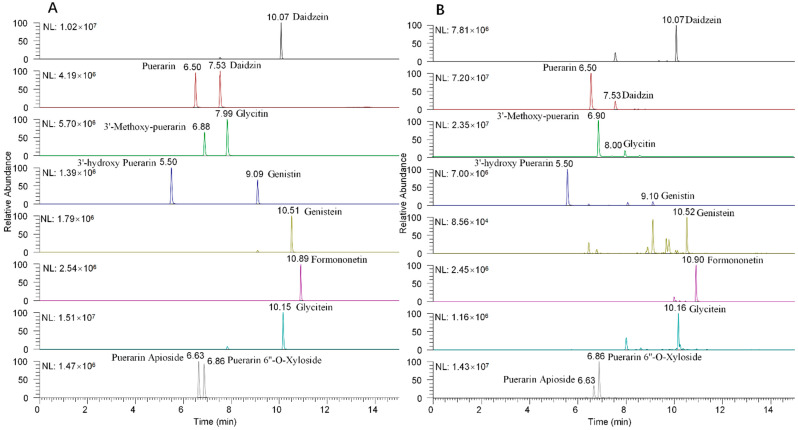
Extracted mass chromatograms of the 12 isoflavones in standard solution (**A**) and sample (**B**).

Isoflavones range from non-polar to medium-polar, so an organic modifier is required to raise the polarity of the mobile phase used for isoflavone analysis [31]. Methanol and acetonitrile were assessed for the chromatographic separation of the 12 isoflavones, with acetonitrile giving the better separation and higher response. Addition of formic acid is also known to improve separation and ionization [32]. Aqueous formic acid solutions (0, 0.05, 0.1, 0.2, and 0.3%) were evaluated for the separation of the isoflavones. Peak shape and sensitivity were maximized for the positively ionized compounds in the presence of 0.2% formic acid. Extracted ion chromatograms for the 12 isoflavones in standard solution (A) and sample matrix (B) are shown in Figure 2.

### 3.2. Optimization of Extraction Conditions

**Figure 3:**
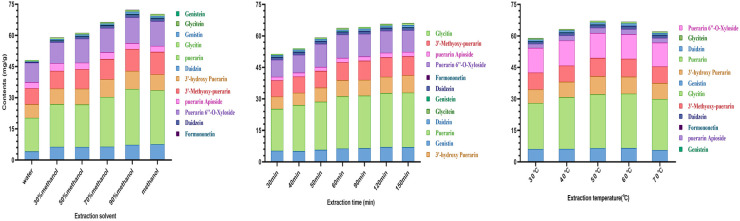
Effect of extraction solvent, temperature, and time on the concentration of isoflavones in pueraria extracts.

Extraction solvent (water and 30, 50, 70, 90, and 100% methanol), time (30 to 150 min), and temperature (30 to 70 °C) were optimized (Figure 3). The concentrations of the 12 isoflavones in the extracts increased when the methanol concentration was increased from 0% to 90% but decreased with 100% methanol. Adding water to organic solvents is known to improve the solubility of polar isoflavones [1]. Isoflavone concentrations increased with longer extraction times but plateaued around 60 min. Isoflavone concentrations were not significantly different in extracts at 50 °C to 60 °C but decreased at 70 °C temperatures. The selected optimum extraction conditions were 90% methanol and ultrasonic extraction for 60 min at 50 °C.

### 3.3. Method Validation

The linear correlation coefficient (r^2^) was greater than 0.998 in all cases. The LODs ranged from 0.10 to 2.78 μg/kg, and LOQs from 0.30 to 9.26 μg/kg. Intra-day precision (RSD) ranged from 1.4 to 6.0%, and inter-day precision from 3.2 to 8.3%. Accuracy (recovery) ranged from 81.5 to 114.8%. These validation data (Table 2) demonstrate that this method is suitable for determining the concentration of the 12 isoflavones compounds in pueraria samples.

### 3.4. Quantitative Analysis of Isoflavones in Pueraria

The developed method was applied to the analysis of 12 isoflavones in six pueraria samples. Average concentrations are summarized in Table 3. Puerarin was present at the highest concentration, followed by puerarin 6″-O-xyloside, 3′-methoxy puerarin, and 3′-hydroxy puerarin. The observed variations in isoflavone concentrations across the pueraria samples may be related to the sample origins and varieties [21].

### 3.5. Qualitative Analysis of Bioactive Components in Pueraria

Bioactive components in pueraria were analyzed using a UHPLC-Q-Orbitrap HRMS technique. A total of 88 compounds were identified, including flavonoids, terpenoids, alkaloids, organic acids, quinones, lipids, phenylpropanoids, and phenols, with the flavonoids being the most abundant small molecules (Table 4).

### 3.6. α-Glucosidase Inhibitory Activity and Molecular Docking

**Figure 4:**
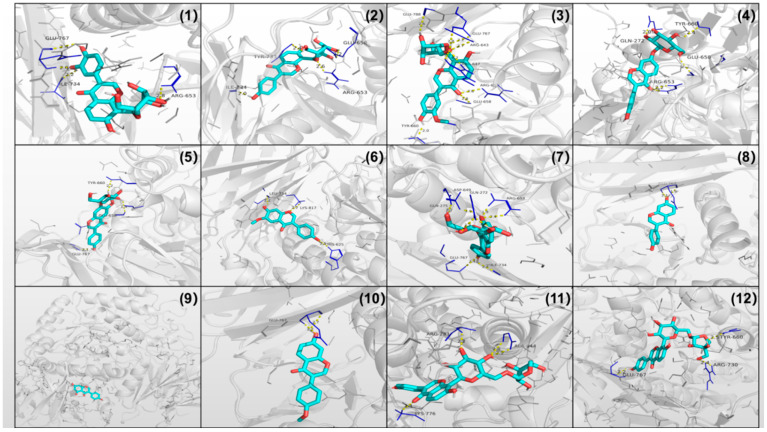
Molecular docking conformations of isoflavones with α-glucosidase (short, dotted yellow lines represent hydrogen bonds. (**1**). 3′-hydroxy puerarin, (**2**). puerarin, (**3**). 3′-methyoxy puerarin, (**4**). daidzin, (**5**). glycitin, (**6**). glycitein, (**7**). genistin, (**8**). daidzein, (**9**). genistein, (**10**). formononetin, (**11**). puerarin 6″-O-xyloside, (**12**). puerarin apioside).

All 12 isoflavones, except for genistein, exhibited α-glucosidase inhibitory activity (Table 5). Daidzin, 3′-methoxy puerarin, and genistin demonstrated slightly lower inhibitory activity than acarbose. Studies have shown that phenolic compounds with isoflavone skeletons exhibit in vitro α-glucosidase inhibition activity [14,33]. Similar to our study, Liu et al. [34] demonstrated, using high resolution α-glucosidase and radical scavenging profiles, that a crude methanol extract of *Pueraria lobata* was a potent α-glucosidase inhibitor. Molecular docking analysis was performed to explore the structure-activity relationships of these compounds (Figure 4) and the findings were consistent with the α-glucosidase inhibition test. The method of molecular docking provides new perspective for the study of active substances in food [35,36]. 3′-Methoxy puerarin was the most potent compound, with a docking energy of −7.9 kcal/mol (the same as the positive control acarbose). 3′-Methoxy puerarin formed nine hydrogen bonds with seven active site residues in the receptor protein. Genistin formed eight hydrogen bonds with six active site residues. However, acarbose formed ten hydrogen bonds with seven active site residues, with a shorter hydrogen bond length. Consequently, acarbose had stronger binding affinity with α-glucosidase than the 12 isoflavones. Previous research has shown that pueraria exerts its antidiabetic activity not only via suppression of α-amylase, α-glucosidase, and the sodium-dependent glucose transporter, but possibly through other means. Pueraria protects against STZ-induced diabetes via antioxidant, antiapoptotic, antihypoxic, and anti-inflammatory pathways [37]. It regulates glucose and lipid metabolism to improve insulin resistance by various means in Luo [38]. Pueraria also increases the expression of glucose transporter type 4 [5]. However, beneficial in vitro properties of compounds should be validated by in vivo studies.

## 4. Conclusions

A rapid and sensitive quantitative method was developed and validated for the simultaneous determination of 12 isoflavones in pueraria using UHPLC-Q-Orbitrap HRMS. The method showed good linearity, accuracy, and precision. In 12 isoflavones, the content of puerarin was the highest. Qualitative analysis also identified 88 small molecule components, with flavonoids being the most abundant. α-Glucosidase inhibition testing and molecular docking elucidated the interactions between the isoflavones and α-glucosidase, confirming the inhibitory activity of these bioactive components. Molecular docking showed that 3′-methoxy puerarin had the highest α-glucosidase inhibitory activity. Pueraria is a promising functional food that could potentially be used as a α-glucosidase inhibitor to control postprandial hyperglycemia. Further in vivo studies are needed to explore the α-glucosidase inhibitory effects of pueraria.

## Figures and Tables

**Table 1:** The detailed MS parameters of 12 compounds.

No.	Compound	CAS	Retention Time	Adduct Ion	Precursor Ion	Delta	Product Ion
			(min)		(m/z)	(ppm)	(m/z)
1	3′-Hydroxy puerarin	117076-54-5	5.50	[M + H]+	433.11365	0.169	313.07080, 283.06024, 415.10297
2	Puerarin	3681-99-0	6.50	[M + H]+	417.11819	0.043	297.07581, 321.07520, 399.10709
3	3′-Methoxy puerarin	117047-07-1	6.88	[M + H]+	447.12888	0.069	327.08643, 429.11844, 297.07590
4	Daidzin	552-66-9	7.53	[M + H]+	417.11811	0.024	255.06538, 199.07590, 227.07002
5	Glycitin	40246-10-4	7.83	[M + H]+	447.12851	−0.013	285.07599, 270.05240, 229.08578
6	Glycitein	40957-83-3	10.15	[M + H]+	285.07571	−0.014	270.05215, 242.05724, 225.05472
7	Genistin	529-59-9	9.09	[M + H]+	433.11301	0.021	271.06015, 128.06224, 153.01804
8	Daidzein	486-66-8	10.07	[M + H]+	255.06535	0.063	227.07027, 199.07556, 137.02327
9	Genistein	446-72-0	10.51	[M + H]+	271.06033	0.085	243.06522, 215.07027, 153.01839
10	Formononetin	485-72-3	10.89	[M + H]+	269.08060	−0.089	254.05652, 213.09102, 197.05974
11	Puerarin 6″-O-xyloside	114240-18-5	6.86	[M + H]+	549.16083	0.102	297.07562, 417.11765, 399.10779
12	Puerarin apioside	103654-50-8	6.63	[M + H]+	549.16052	0.046	297.07556, 417.11746, 399.10742

**Table 2:** Results of Linear regression, LOD, LOQ, recovery test and precision of 12 compounds.

Compounds	Linear Regression Data		LOD	LOQ	Recovery Test				Precision (RSD, %, *n* = 6)	
	Equation	Linearity (r^2^)	(μg/kg)	(μg/kg)	Originals (μg)	Spiked (μg)	Found (μg)	Recovery (%)	Intra-Day	Inter-Day
3′-Hydroxy puerarin	Y = 48,674.5X + 2400.93	0.9994	1.83	6.10	130.1	65.0	193.4	97.4	2.2	3.6
						130.0	259.3	99.4	2.8	5.2
						195.0	349.7	102.6	1.4	4.9
Puerarin	Y = 118,793X + 339,229	0.9991	0.57	1.89	402.8	200.0	555.4	96.3	1.7	5.3
						400.0	741.7	104.7	2.8	5.1
						600.0	904.6	93.6	1.9	4.8
3′-Methoxy puerarin	Y = 110,435X + 194,781	0.9989	0.95	3.16	158.4	80.0	223.6	91.5	2.9	4.1
						160.0	281.1	96.7	3.0	5.6
						240.0	358.8	103.5	2.2	5.3
Daidzin	Y = 127,342X + 344,625	0.9987	0.63	2.08	108.9	54.0	150.0	90.1	2.3	5.9
						108.0	190.0	95.1	2.7	4.2
						162.0	242.0	92.2	1.8	5.4
Glycitin	Y = 182,859X + 539,463	0.9990	0.57	1.89	12.4	6.0	17.0	86.2	3.2	7.3
						12.0	21.4	85.2	3.8	5.1
						18.0	32.4	91.0	2.6	5.5
Glycitein	Y = 384,176X + 499,669	0.9995	0.12	0.40	2.0	1.0	3.0	88.6	5.8	7.5
						2.0	4.3	112.2	4.2	5.9
						3.0	5.4	114.8	3.5	5.4
Genistin	Y = 30,610.1X – 34,832.6	0.9989	2.78	9.26	16.6	8.0	23.0	92.9	3.4	6.0
						16.0	29.1	88.1	3.8	7.3
						24.0	35.5	95.8	3.2	6.7
Daidzein	Y = 246,022X + 456,106	0.9992	0.18	0.61	20.0	10.0	27.7	87.0	3.4	5.6
						20.0	36.4	89.9	3.7	4.8
						30.0	50.0	100.1	2.2	4.2
Genistein	Y = 49,834.2X – 66,499.2	0.9991	0.95	3.16	1.4	0.7	2.0	81.5	5.6	8.3
						1.4	3.0	90.8	5.3	6.6
						2.1	3.9	89.9	3.5	3.2
Formononetin	Y = 614,878X + 47,444.8	0.9993	0.10	0.30	1.1	0.6	1.7	89.4	6.0	6.4
						1.2	2.2	94.4	3.7	7.0
						1.8	2.8	90.6	5.5	5.9
Puerarin 6″-O-xyloside	Y = 42,977.4X + 57,239.4	0.9996	1.88	6.25	226.4	110.0	309.2	95.3	2.1	4.6
						220.0	398.9	103.4	2.6	5.2
						330.0	497.0	92.0	1.8	3.8
Puerarin apioside	Y = 45,781.5X + 38,439.8	0.9994	1.63	5.43	60.0	30.0	89.1	97.0	4.0	5.8
						60.0	116.1	93.5	2.8	6.2
						90.0	138.4	87.1	3.3	7.0

**Table 3:** The content of the 12 isoflavones compounds in 6 pueraria samples (mg/g).

No.	Compound	Sample 1	Sample 2	Sample 3	Sample 4	Sample 5	Sample 6
1	3′-Hydroxy puerarin	10.56 ± 1.21	7.70 ± 2.03	8.52 ± 0.94	7.66 ± 1.79	10.81 ± 0.84	8.67 ± 2.02
2	Puerarin	27.6 ± 2.80	17.68 ± 1.52	22.42 ± 0.80	16.70 ± 3.80	19.00 ± 0.92	25.20 ± 1.61
3	3′-Methoxy-puerarin	9.83 ± 0.83	10.43 ± 2.12	8.10 ± 1.25	6.90 ± 1.45	9.91 ± 2.32	10.25 ± 3.10
4	Daidzin	6.53 ± 1.00	6.59 ± 0.34	5.31 ± 0.67	5.39 ± 1.21	5.82 ± 0.53	7.05 ± 0.63
5	Glycitin	0.95 ± 0.23	1.04 ± 0.08	0.83 ± 0.18	0.73 ± 0.04	1.02 ± 0.32	0.83 ± 0.33
6	Glycitein	0.23 ± 0.05	0.09 ± 0.04	0.30 ± 0.10	0.12 ± 0.04	0.14 ± 0.02	0.09 ± 0.01
7	Genistin	1.15 ± 0.03	1.04 ± 0.17	0.86 ± 0.32	0.92 ± 0.09	1.07 ± 0.04	1.17 ± 0.05
8	Daidzein	0.94 ± 0.26	0.64 ± 0.09	1.47 ± 0.15	0.80 ± 0.32	0.79 ± 0.08	1.40 ± 0.10
9	Genistein	0.05 ± 0.02	0.03 ± 0.01	0.09 ± 0.03	0.05 ± 0.03	0.05 ± 0.02	0.09 ± 0.04
10	Formononetin	0.03 ± 0.01	0.04 ± 0.02	0.06 ± 0.02	0.04 ± 0.03	0.06 ± 0.02	0.07 ± 0.02
11	Puerarin 6″-O-xyloside	9.07 ± 0.73	8.15 ± 1.54	7.62 ± 0.98	8.70 ± 2.01	7.47 ± 0.83	13.18 ± 2.39
12	Puerarin apioside	1.23 ± 0.05	1.04 ± 0.13	0.93 ± 0.28	1.28 ± 0.16	0.93 ± 0.21	2.78 ± 0.06

**Table 4:** Typical compounds identified from Pueraria by UHPLC-Q-Exactive Orbitrap/MS.

No.	Compound	Formula	RT	Adduct Ion	Precursor Ion	Delta	Product Ion
			(min)		(m/z)	(ppm)	(m/z)
1	Guanine	C5H5N5O	0.76	[M + H]+	152.0567	0.077	110.03531, 135.03018
2	L-Tyrosine	C9H11NO3	0.87	[M + H]+	182.08121	0.205	136.07578, 123.04443
3	5-Hydroxymethylfurfural	C6H6O3	0.87	[M + H]+	127.03912	1.149	81.0342, 109.02873
4	L-Phenylalanine	C9H11NO2	0.92	[M + H]+	166.08615	−0.656	120.08102, 149.05966
5	Diglycolic acid	C4H6O5	0.94	[M − H]−	133.01241	−0.74	72.99128, 89.02254
6	Adenosine	C10H13N5O4	1.15	[M + H]+	268.10385	−0.666	136.06184, 85.02911
7	5-Hydroxymethylfurfural	C6H6O3	1.18	[M + H]+	127.03904	0.548	81.03403, 109.02885
8	Vitexin7-O-sulfate	C24H15O13	2.35	[M − H]−	511.05307	2.353	421.02237, 391.01184, 283.06030, 262.00897
9	Diethyl tartrate	C8H14O6	4.26	[M − H]−	205.07042	−0.245	72.99133, 115.07466, 129.05391, 143.06963
10	3,5,7-Trihydroxyflavone 3-glucoside−8-sulfate	C24H15O13	4.73	[M − H]−	511.05325	2.533	311.05515, 391.01157, 283.06018, 341.06555
11	3′-Hydroxy−4′-O-β-D-glucosyl-Puerarin	C27H30O15	5.01	[M + H]+	595.16559	−0.157	313.07053, 283.06000, 433.11307
12	Puerarin−4′-O-β-D-glucopyra noside	C27H30O14	5.42	[M + H]+	579.17059	−0.237	267.06497, 297.07559, 417.11777
13	Hypaphorine	C14H18N2O2	5.6	[M + H]+	247.144	0.063	188.07057, 118.06542, 146.06004
14	7,8-Dihydroxyflavone	C15H10O4	6.24	[M + H]+	255.06511	−0.075	227.07043, 237.05447, 199.07541
15	3′-Hydroxy Puerarin *	C21H20O10	6.42	[M + H]+	433.1127	−0.223	313.07047, 283.05994, 433.11270
16	Apigenin-6-C-glucoside-7-O-glucoside	C27H30O15	6.64	[M − H]−	593.14929	−0.806	473.10712, 310.04730, 282.05231
17	3′-Methoxy-4′-O-glucosyl-Puerarin	C28H32O15	6.71	[M + H]+	609.18152	0.123	285.07562, 270.05203
18	3′-Hydroxy puerarin xyloside	C26H28O14	7.06	[M + H]+	565.15515	−0.032	313.07043, 283.05981, 433.11282
19	Puerarin xyloside	C26H28O13	7.29	[M − H]−	547.1438	−0.817	267.06537, 295.06003, 275.07001
20	Puerarin-6″-O-glucoside	C27H30O14	7.39	[M + H]+	579.17053	−0.302	267.06500, 297.07562, 399.10739
21	Puerarin *	C21H20O9	7.45	[M + H]+	417.11768	−0.329	297.07559, 267.06503, 399.10727
22	Puerarin-6″-O-apioside *	C26H28O13	7.97	[M + H]+	549.15997	−0.297	417.11771, 267.06500, 297.07559
23	Puerarin apioside *	C26H28O13	8.03	[M + H]+	549.15985	−0.763	297.07568, 381.09705
24	Daidzein-6-C-(6″-glucosyl)glucoside	C27H30O14	8.15	[M + H]+	579.17059	−0.242	327.04984, 299.05505
25	3′-Methoxy puerarin *	C22H22O10	8.6	[M + H]+	447.12842	−0.153	327.08609, 297.07550, 429.11783
26	Daidzin *	C21H20O9	8.62	[M + H]+	417.1174	−0.609	255.06500, 199.07533, 227.07014
27	Calycosin	C16H12O5	8.66	[M − H]−	283.06033	−3.062	268.03683, 211.03873
28	3,2′-Dihydroxyflavone	C15H10O4	8.67	[M − H]−	253.04967	0.135	224.04665, 133.02780
29	Genistein-4′-O-glucoside	C21H20O10	9.01	[M + H]+	433.11108	−1.843	313.07007, 283.06003, 415.10181
30	Genistein-8-C-glucoside	C21H20O10	9.09	[M + H]+	433.11276	−0.163	313.07047, 283.05994, 415.10199
31	Glycitin *	C22H22O10	9.12	[M + H]+	447.12952	0.947	285.07562, 270.05206, 253.04936
32	3′-Methoxydaidzein	C16H12O5	9.14	[M + H]+	285.07556	−0.19	285.07556, 270.05200, 253.04945
33	Genistein-8-C-(6″-O-apioside)-glucoside	C26H28O14	9.21	[M + H]+	565.1543	−0.877	433.11264, 313.07028, 283.05978
34	Apigenin	C15H10O5	9.4	[M + H]+	271.05966	−1.616	215.07030, 153.01831, 243.06546
35	Thermopsoside	C22H22O11	9.62	[M − H]−	461.1076	−0.198	298.04742, 341.06580, 326.04242
36	Oroxin B	C27H30O15	9.62	[M − H]−	593.14978	−0.866	269.04340, 341.06537, 298.04712
37	Daidzein-4′-O-glycoside	C21H20O9	9.64	[M + H]+	417.11743	−0.579	255.06497, 199.07524, 255.06497
38	Pueroside A	C29H34O14	9.67	[M + H]+	607.2017	−0.21	107.04955, 299.0919, 253.08572
39	Genistein-8-C-apiosyl (1-6)- glucoside	C26H28O14	10.05	[M + H]+	565.15411	1.072	271.05994, 433.11292
40	Genistin	C21H20O10	10.25	[M + H]+	433.1123	−0.623	215.07022, 153.01828, 271.06003
41	Emodin	C15H10O5	10.25	[M-H]-	269.04468	0.23	224.04639, 133.02776
42	Isoembigenin	C23H24O10	10.37	[M + H]+	461.144	−0.388	107.04961, 299.09137, 253.08554
43	Salicylic acid	C7H6O3	10.65	[M − H]−	137.02267	−0.651	137.02267, 93.03277
44	6″-O-Malonyl daidzin	C24H22O12	10.66	[M + H]+	503.11694	−1.462	255.06517, 199.07542
45	Kaempferide	C16H12O6	10.93	[M − H]−	299.05505	0.035	284.03162, 255.02873, 299.05505
46	Formononetin-8-C-glucosid e-O-xyloside	C27H30O13	11	[M + H]+	563.17535	−0.567	431.13345, 311.09109, 281.08060
47	Biochanin A *	C16H12O5	11.1	[M − H]−	283.06015	0.05	268.03677, 239.03368, 211.03868
48	5-Hydroxy genistein-4′-O-(6″-malonyl) glucoside	C25H24O13	11.12	[M + H]+	533.12976	0.793	285.07574, 270.05219
49	Azelaic acid	C9H16O4	11.63	[M − H]−	187.09612	−0.385	126.09541, 187.09610
50	6″-O-Acetyl Daidzin	C23H22O10	11.85	[M + H]+	459.12704	−1.533	255.06511, 199.07536
51	13-HODE	C18H32O3	11.93	[M − H]−	295.22656	−0.211	277.21674, 195.13684, 224.59799
52	Ferulic Acid	C10H10O4	11.93	[M − H]−	193.04945	−0.085	134.03557, 178.02600
53	Genistein-4′-O-(6″-malonyl)glucoside	C24H22O13	12.14	[M + H]+	519.11395	0.633	215.07027, 153.01833, 271.06012
54	Curcumenol	C15H22O2	12.57	[M − H]−	233.15355	−0.056	214.91223, 119.00460
55	Chrysin	C15H10O4	12.88	[M − H]−	253.04951	−0.025	224.04666, 209.05945
56	Daidzein *	C15H10O4	12.93	[M + H]+	255.06516	−0.025	199.07542, 227.07018, 137.02342
57	Glycitein	C16H12O5	13.45	[M + H]+	285.07574	−0.01	285.07574, 270.05222, 253.04958
58	Ononin	C16H12O5	13.68	[M + H]+	431.1364	−0.019	269.08075, 293.08151
59	2″,6″-Di-O-Acetyl isovitexin	C22H22O9	14.54	[M + H]+	517.13501	0.957	268.08087, 254.05725
60	Adenine	C20H16O4	14.54	[M + H]+	136.0618	0.028	119.03558, 91.05481
61	Tournefolal	C5H5N5	15.17	[M − H]−	269.04456	0.11	133.02759, 224.04640
62	Genistein	C15H10O5	15.25	[M + H]+	271.05997	−0.13	153.01831, 215.07030, 243.06546
63	Fraxetin	C15H10O5	15.25	[M + H]+	209.19009	0.098	167.14314, 153.12741, 111.08086
64	D-Gluconic acid	C10H8O5	15.35	[M − H]−	195.0495	−0.429	159.02824, 129.01747
65	Guanine	C6H12O7	15.6	[M + H]+	152.05678	0.094	128.04552, 110.03531, 135.03018
66	Formononetin-7-O-(6″-acetylglucoside)	C5H5N5O	16.08	[M + H]+	473.14249	−1.733	269.08066, 254.05722
67	Schaftoside	C24H24O10	16.64	[M − H]−	563.13873	−0.802	311.05511, 283.06024, 133.02768
68	Isoferulic acid	C26H28O14	17.09	[M − H]−	193.04945	−0.085	134.03557, 149.05949
69	Formononetin *	C10H10O4	17.62	[M + H]+	269.08072	−0.79	213.09091, 118.04166
70	Pterolactam	C16H12O4	17.63	[M + H]+	116.07082	0.215	70.06588, 99.01923
71	Dimethyl-1-Phenylazulene-4,5-dicarboxylate	C5H9NO2	19.92	[M + H]+	321.11185	−0.286	147.04407, 159.08043, 306.08713
72	4-Hydroxybenzaldehyde	C20H16O4	19.93	[M + H]+	123.04082	−3.236	118.03519, 100.02482, 82.01437
73	Licoflavone A	C7H6O2	21.06	[M + H]+	323.12753	−0.256	267.06509, 239.07011, 199.07509
74	5-Hydroxy-6,7-dimethoxyflavone	C20H18O4	21.09	[M − H]−	297.07553	−0.215	282.05240, 267.02899, 239.03381
75	Neobavaisoflavone	C17H14O5	21.1	[M − H]−	321.11197	−0.166	237.05444, 265.04950, 277.04938
76	Isoliquiritigenin	C20H18O4	21.1	[M + H]+	257.08066	−0.175	137.02339, 147.04402, 119.04937
77	Glabrone	C15H12O4	23.5	[M − H]−	335.09191	0.21	307.09659, 291.06564, 277.04761
78	Oleamide	C20H16O5	23.63	[M + H]+	282.27893	−0.211	265.25244, 247.24191, 69.07063, 83.08614
79	Stigmasterol	C18H35NO	23.72	[M + H]+	413.37805	0.257	109.06524,97.06529, 395.36667
80	Physcion	C29H48O	23.73	[M − H]−	283.06024	0.14	268.03671, 239.03365, 211.03847
81	β-Sitosterol	C16H12O5	26.98	[M + H]+	415.72112	4.522	91.05481, 384.09723
82	Isoliquiritigenin	C10H14O	26.99	[M − H]−	255.06522	0.035	119.04842, 135.00705, 153.01749
83	Proline	C5H9NO2	27.56	[M + H]+	116.07082	0.215	70.06588, 99.01923, 73.04050
84	Allantoin	C4H6N4O3	27.69	[M − H]−	157.03476	−0.857	114.02900, 111.95873
85	Glyinflanin G	C25H24O5	29.59	[M + H]+	405.16962	−0.03	281.0444, 319.09567, 293.04517
86	Psoralidin	C20H16O5	29.59	[M + H]+	337.10654	−0.51	309.11124, 281.11731, 253.05052, 223.07600
87	Curcumanolide A	C15H22O2	35.42	[M + H]+	235.16924	−0.016	179.10669, 123.04434
88	7-Methoxycoumarin	C10H8O3	38.04	[M + H]+	177.05463	0.009	63.03896, 149.05975, 135.04413, 117.03380

The compounds marked with “*″ were identified by the reference standards.

**Table 5:** The result of α-glucosidase inhibitory activity (IC_50_) and molecular docking.

No.	Compound	IC_50_	Docking Energy	Active Site Residues	Hydrogen Bond Number
		(mg/L)	(kcal/mol)		
1	3′-Hydroxy puerarin	203.6 ± 4.2	−8.6	GLU-767, ILE-734, ARG-653	4
2	Puerarin	154.2 ± 1.7	−8.1	GLU-658, ILE-734, ARG-653, TYR-733	4
3	3′-Methoxy-puerarin	95.9 ± 3.8	−7.9	GLU-767, GLU-658, ARG-643, ARG-647, ARG-653, TYR-660, GLU-788	9
4	Daidzin	72.94 ± 2.5	−8.3	GLU-658, GLN-272, ARG-653, TYR-660	5
5	Glycitin	139.7 ± 5.1	−9.0	GLU-658, GLU-767, TYR-660	3
6	Glycitein	246.0 ± 2.6	−8.1	LEU-754, LYS-817, HIS-625	3
7	Genistin	101.3 ± 6.3	−8.7	GLN-275, ASP-649, GLN-272, ARG-653, GLU-767, ILE-734	8
8	Daidzein	114.8 ± 1.9	−8.1	GLU-767	2
9	Genistein	/	−8.1	/	/
10	Formononetin	128.3 ± 2.0	−8.1	GLU-767	2
11	Puerarin 6″-O-xyloside	169.6 ± 3.4	−8.7	LYS-776,ARG-283,ALA-644	4
12	puerarin apioside	96.55 ± 2.6	−9.0	GLU-767,TYR-660,AGR-730	3
13	Acarbose	58.6 ± 4.4	−7.9	GLN-275,ASP-759,GLN-272,ARG-730,GLU-662,TYR-660,GLY-731	10

## Data Availability

Data are contained within the article.
